# An Ecofriendly synthesis of silver nano-bioconjugates by *Penicillium citrinum* (MTCC9999) and its antimicrobial effect

**DOI:** 10.1186/2191-0855-3-16

**Published:** 2013-02-23

**Authors:** Achintya Mohan Goswami, Tuhin Subhra Sarkar, Sanjay Ghosh

**Affiliations:** 1 Department of Biochemistry, University of Calcutta, 35, Ballygunge Circular Road, Kolkata, West Bengal 700 019, India; 2 Department of Physiology, Krishnagar Government College, Krishnagar, Nadia, West Bengal PIN-74101, India

**Keywords:** Green synthesis, *Penicillium citrinum*, Transmission Electron Microscopy (TEM), Zeta potential, Fourier Transformed Infra-Red Spectroscopy (FTIR), Minimum Inhibitory Concentrations (MIC), Minimum Bactericidal Concentrations (MBC), Reactive Oxygen Species (ROS)

## Abstract

This report provides for the first time a novel environment friendly extracellular synthesis of stable silver nano-bioconjugates (SNBCs) at room temperature at pH 5.0 using *Penicillium citrinum* MTCC 9999 biomass. The UV-Visible spectral scan of dispersed SNBCs solution showed absorption in the region 340–450 nm due to surface plasma resonance (SPR). Typical Transmission Electron Microscopic (TEM) images showed that although two populations were present but most of them were in 20–30 nm range. Average zeta potential of SNBCs was −21 mV suggesting some biomolecules capped the nanoparticles imparting a net negative charge over it. FTIR analysis also showed that biomolecules were involved in stabilization. SNBCs showed strong antibacterial activity against both Gram positive (*Bacillus subtilis*) and Gram negative bacteria (*Escherichia coli*). SNBCs also showed strong antifungal activity as assessed against *Schizosaccharomyces pombe*. In the case of *E. coli* the minimum inhibitory concentrations (MIC) of SNBCs was 4 μg/ml while in *B. subtilis* it was 8 μg/ml. In the case of *E. coli* the minimum bactericidal concentrations (MBC) of SNBCs was 8 μg/ml while in *B. subtilis* it was 32 μg/ml. The SNBCs exerted its antibacterial and antifungal activity through generation of reactive oxygen species (ROS) inside the cell.

## Introduction

The field of nanotechnology has got major advances in various aspects of technology from biosensors to medicine ( Velev and Kaler 1999; Nie and Emory 1997; Gu et al. 2003; Kim et al. 2008). Various optic based analytical techniques are designed based on the surface plasma resonance (SPR) properties of silver nanomaterials ( Lee and EI-Sayed 2006). There is also growing interest in biomedical applications of silver nanoparticles ( Sun et al. 2005). But synthesis of nanoparticles requires harsh reducing agents (e.g. sodium borohydride, hydroxyl amine), capping agents (e.g. trioctylphosphine oxide) and organic solvents (e.g. toluene, chloroform) as well as high temperature and pressure ( Xie et al. 2007). So the focus is turned on to the environmental friendly synthesis of nanoparticles, the so called “Green Chemistry” ( Klaus et al. 1999; Raveendran et al. 2003). Complete green synthesis of silver nanoparticles requires environmentally acceptable solvent, eco friendly reducing and capping agents. In all these respects biological approach is more convenient.

A number of biological species has been shown to produce silver nanoparticles either intracellular or cell surface based or extracellular. A study with *Pseudomonas stutzeri* AG259, a metal accumulating bacteria have been shown to synthesize silver nanoparticles in the periplasmic space with a size ranging from a few nanometers to 200 nm of different shapes and morphologies (spherical, triangular, truncated triangular) ( Klaus et al. 1999). *Lactobacillus* strains have been shown to synthesize silver nanoparticles and these form clusters on the cell surface ( Nair and Pradeep 2002). Vigneshwaran N et al. showed a cell-surface based synthesis of silver nanoparticles with a varying particle size from 4–14 nm by *Aspergillus flavus* (Vigneshwaran et al. 2007a). Extracellular biosynthesis of silver nanoparticles of 5–25 nm diameter by *Aspergillus fumigatus* has been studied (Bhainsa and D’Souza and *Penicillium fellutanum*^2006^; Kathiresan et al. 2009(Monali et al. ). Polydisperse silver nanoparticles of 20–60 nm diameter are synthesized extracellularly by *Alternaria alternata*^2009^). Apart from using microorganisms as a factory of synthesizing nanomaterials, soluble starch has been used in the ecofriendly synthesis of silver nanoparticles with a size 23–35 nm ( Vigneshwaran and Nachane 2006). Spent mushroom substrate (SMS) has also been used as a simple root for the synthesis of silver-protein (core- shell) nanoparticles having average size around 30 nm ( Vigneshwaran A and Kathe 2007).

There is also increasing demand for finding antimicrobial agents due to ever increasing bacterial resistance to antibiotics and consequent development of multidrug resistance in bacteria. Recently nanoparticles have been successfully used for the delivery of therapeutic agents (Zhang et al. 2008a), in chronic disease diagnostics ( Hong et al. 2008), to reduce bacterial infections in skin and burn wounds ( Rai et al. 2009), to prevent bacterial colonization on medical devices and in the food and clothing industries as an antimicrobial agent ( Chau et al. 2007; Vigneshwaran et al. 2007b). For centuries, silver is known for its antimicrobial activity against a diverse group of bacteria and has been used for many years as an antimicrobial substance ( Castellano et al. 2007). Silver nanoparticles have been shown to have potent antibacterial, antifungal and antiviral activities. Compared with other metals, silver nanoparticles show higher toxicity to microorganisms while exhibiting lower toxicity to mammalian cells ( Zhao and Stevens 1998). A large number of researches were carried out to investigate the bactericidal activity of silver nanoparticles. Silver nanoparticles interact with gram-negative bacteria in a size dependent fashion ( Sukdeb et al. 2007). It has been suggested that the antibacterial activity is due to silver ions, released from metallic bulk silver or from nanoparticle (NP) surfaces, which interact with the thiol groups in bacterial proteins or interfere with DNA replication ( Feng et al. 2000; Wu et al. 2009). It has also been reported that silver ions can affect the respiratory chain in bacteria ( Holt and Bard 2005). On the other hand, other authors have suggested that silver nanoparticle toxicity may arise directly from physical processes caused by nano-objects, like disruption of cell membrane and penetration of NPs into the cytoplasm ( Xu et al. 2004). So, the scientific debate is still open concerning the mechanism of the antibacterial effect of silver nanoparticles ( Sondi and Salopek-Sondi 2004; Dror-Ehre et al. 2009; Zhang et al. 2008b). It was reported that silver nanoparticles stabilized with sodium dodecyl sulfate (SDS) have no antibacterial activity because the negatively charged SDS interferes with the absorption of microbes to the surface of the nanoparticles or silver ions ( Cho et al. 2005). So the application of silver nanoparticles as antimicrobial agent requires appropriate coating of nanoparticle surface to avoid aggregation and to favour solubility in watery environment and attachment of nanoparticle to bacterial cell surface.

Until now, little research has been done on the antimicrobial activity of biologically produced nanosilver and its specific mode of action. The aim of this study was to examine the antimicrobial properties of biogenic silver. In the present work, we report an environment friendly procedure for synthesis of stable silver nano-bioconjugate (SNBC) and its potential application as antimicrobial agent. We prefer to use the term nano-bioconjugate because of the presence of biomolecules on the nanoparticle surface, which are used for further studies without any further chemical modification of nanoparticle surface.

## Materials and methods

All chemical reagents were purchased from Sigma (St. Louis, MO, USA) and were of analytical grade. All components for growth media were purchased from Becton–Dickinson (Rutherford, NJ, USA).

### Microorganism

*Penicillium citrinum* (strain number MTCC 9999) was isolated in our laboratory from soil, collected from Dhapa situated near Kolkata, West Bengal. The strain was sent for identification to the Institute of Microbial Technology (IMTECH), Chandigarh, India, a centre for microbial strain identification and maintenance. The strain was identified as *Penicillium citrinum* by them and it was deposited in the IMTECH strain bank. The strain was subcultured on potato dextrose agar (PDA).

### Biomass production

The fungus (*P. citrinum* MTCC 9999) was grown aerobically in liquid media containing (g/l) KH_2_PO_4_: 7.0, K_2_H PO_4_:2.0, MgSO_4_, 7H_2_O:0.1, (NH_4_)_2_SO_4_:1.0, yeast extract: 0.6, glucose 10.0. The conical flask containing the above sterilized media was inoculated with fungal spores and incubated at orbital shaker at 29°C for 84 hours at 140 rpm. Then the biomass was harvested by sieving through a plastic filter and washed several times with Milli-Q deionized water to remove any traces of media components. Biomass was placed in Mili Q water to collect the fungal cell surface biomolecule or any secretory materials which could have reducing power for the biological synthesis of nanoparticles. Typically 20 g biomass (fresh weight) was dispersed in 200 ml of deionized Milli-Q water. It was then kept for 72 hours at 25°C at 120 rpm in an orbital shaker. After the incubation, cell filtrate was obtained by passing it through Whatman filter paper no1 for the synthesis of silver nano-bioconjugates by extracellular filtrate. Each experiment was repeated thrice using freshly grown culture of *P. citrinum* in PDA.

### Synthesis of silver nano-bioconjugates by extracellular filtrate

Silver nitrate (AgNO_3_) at a final concentration of 0.5 mM was added from a higher stock of 200 mM to the cell filtrate and agitated at 100 rpm in dark at 25°C. Control set (only cell filtrate) without AgNO_3_ was also run side by side. Another negative control containing only 0.5 mM AgNO_3_ were maintained under the same conditions. Silver nano-bioconjugates were characterized by visual inspection. Sample was withdrawn at various time intervals for recording of UV-Visible spectra. UV-Visible spectra were recorded spectrophotometer (V-530) (JASCO Analytical Instruments, 28600 Mary’s Court, Easton, MD 21601).

### Characterization of silver nano-bioconjugates

Dynamic light scattering (DLS) analysis was performed in Zetasizer (MALVERAN Nano Series, Malvern Instruments Ltd, Enigma Business Park, Grovewood Road, Malvern, Worcestershire, UK. WR14 1XZ), to measure the hydrodynamic diameter and zeta potential of SNBCs.

### Transmission electron microscopic (TEM) measurement

The samples for transmission electron microscopy (TEM) analysis were prepared by drop-casting the SNBCs solution on a carbon-coated copper TEM grid. Before casting to the grid the SNBCs solution was centrifuged at 10000 rpm for 5 minutes and the isolated SNBCs were dispersed in 100 μl double distilled water and sonicated in a bath sonicator for 15 minutes. The TEM images were recorded on a high resolution electron microscope (HRTEM: JEOL JEM 2010) operating at an accelerating voltage of 200 kV. Fast Fourier transform (FFT) images were recorded with built-in software for the FFT algorithm for image processing in HRTEM: JEOL JEM 2010 instrument.

### Fourier transformed infra red spectroscopy (FTIR)

For FTIR spectrum analysis the SNBCs were centrifuged at 10,000 rpm for 10 minutes to remove free proteins or other compounds present in the solution. The SNBCs then resuspended in double distilled water and again centrifuged. The process was repeated for three times and finally the centrifuged part containing SNBCs were redispersed in double distilled water and subjected to FTIR spectroscopy.

### Antimicrobial activity of silver nano-bioconjugates (SNBCs)

Antimicrobial activities of SNBCs were assayed by cup-plate method. Antimicrobial activity was assayed against following Gram positive bacteria *Bacillus subtilis* ATCC 6633*,* and Gram negative bacteria *Escherichia coli* ATCC 8739 and fungus *Schizosaccharomyces pombe* ATCC 24843. Zone of inhibition was determined by measuring the diameter of bacterial clearance after 24 hour. Minimum inhibitory concentrations (MIC) and minimal bactericidal concentration (MBC) of the SNBCs were determined following the guidelines of National Committee for Clinical Laboratory Standards ( NCCLS, Approved standards M7–A4 1997). Colony-forming unit (CFU) is an estimate of viable bacterial and fungal (yeast) growth measurement. The Spread Plate technique was used to determine the CFU. Bacterial suspensions were diluted in sterile Muller Hington Broth (MHB) to obtain a final inoculum of 10^6^ CFU/ ml. The concentrations of SNBCs tested were 1, 2, 4, 8, 16, 32, 64 μg/ml. The samples were then incubated at 37°C at 140 rpm for 24 hours. We have also used a control set where *P. citrinum* biomass was placed in Mili-Q water under similar experimental condition like the silver nanobioconjugate production. Minimum inhibitory concentration was determined using the control fungual ell exudates, control 0.5 mM silver nitrate as well as the silver nanobioconjugates.

After incubation, minimum inhibitory concentrations (MIC) were read visually; all samples were plated to nutrient agar and incubated. The minimal bactericidal concentration (MBC) was defined as a 99.9% reduction in CFU from the starting inoculum after 24 h incubation interval. The minimal fungicidal concentration (MFC) was defined as a 99.9% reduction in CFU from the starting inoculum after 24 h incubation interval. Fungal strains of *S. pombe* were grown up in YES medium (0.5% yeast extract, 3% dextrose with proper supplements) at 32°C.

### Fluorescence imaging of reactive oxygen species (ROS) production

Radical production was quantified by the addition of the non-fluorescent precursor molecule 2^′^, 7^′^ dichlorodihydrofluorescein diacetate (DCFDA). In the presence of ROS, DCFDA is oxidized to a fluorescent molecule. Bacterial cells were grown in MHB up to 0.24 O.D. at 590 nm. Fungal strains of *S. pombe* were grown up in YES media up to 0.4 OD at 590 nm. The cells were then centrifuged at 5000 rpm for 5 minutes and cell pellet is collected and dispersed in 50 mM potassium phosphate buffer. And cells were washed to remove media. The cells were then again re-dispersed in 50 mM potassium phosphate buffer at a final cell O.D. of 0.5 at 600 nm. The cells were then treated with SNBCs at a final concentration of 40 μg/ml for 1 hour and 2 hour. After treatment DCFDA was added to the cells at a final concentration of 10 μM.

## Results

Extracellular filtrate (pH 5.0) collected from *P. citrinum* was able to reduce silver nitrate (0.5 mM) to form silver nano-bioconjugates (SNBCs) resulting in the appearance of the brown color indicating the presence of colloidal silver particles in SNBCs ( Burda et al. 2005). However, no change in colour was observed in control sets (Additional file 1 Online Resource 1).

**Figure 1:**
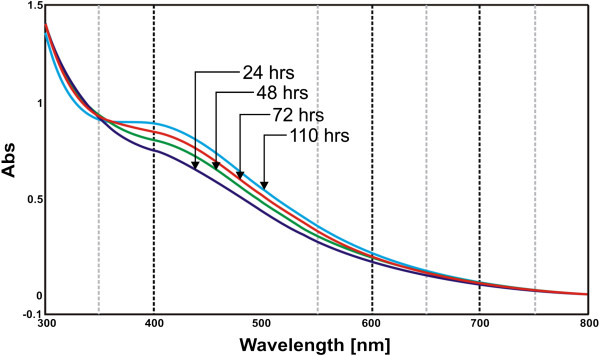
UV-visible spectral scan of SNBCs by bio-reduction of silver nitrate in aqueous solution were recorded at various time points viz. 24 hours, 48 hours, 72 hours and 110 hours in Jasco UV-Visible spectrophotometer (V-530) operated at a resolution of 1 nm in absorption mode.

A time course study was conducted to follow the synthesis of SNBCs (Figure 1) by the extracellular filtrate of pH 5.0. Similar study was conducted with extracellular filtrate of pH 3.0, pH 7.0 and pH 9.0 (Data not shown). All the conditions at different pH generated SPR signal in the region 340–450 nm of SNBCs by UV-visible spectroscopy. An increase in absorbance in the region 340–450 nm with time indicated the synthesis of SNBCs.

**Figure 2:**
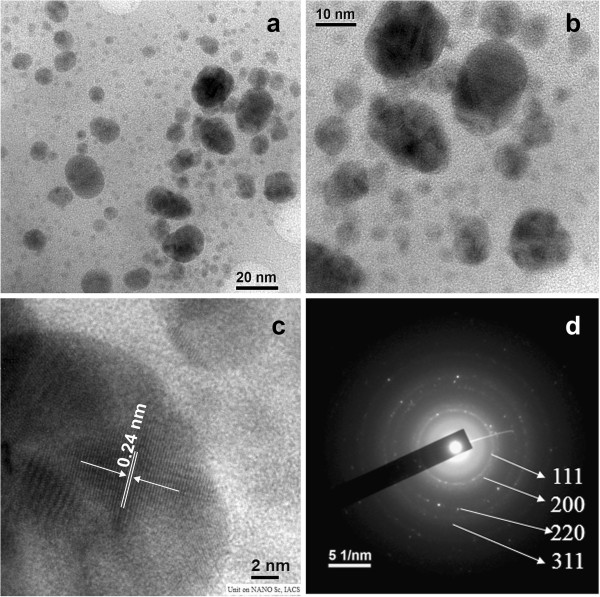
**Characterization of SNBCs by TEM.** (**a**) Typical TEM image of SNBCs. (**b**) A magnified TEM image. (**c**) HRTEM image of SNBCs. (**d**) SAED pattern of SNBCs which were indexed according to (111), (200), (220), and (311) reflections of *fcc* silver on the basis of their *d*-spacings of 2.47 A^0^, 2.13A^0^, 1.49 A^0^, and 1.27 A^0^.

SNBCs were analyzed by Dynamic Light Scattering to measure hydrodynamic diameter immediately after synthesis (Additional file 2 Online Resource 2). Interestingly, SNBCs synthesized by extracellular filtrate of pH 5.0 showed a single population whose hydrodynamic diameter was centered on approximately 20–40 nm. As in dynamic light scattering small particles could be masked by the large particles, so the Z average was 76.42 ± 6.12 nm. SNBCs were further analyzed by TEM to understand the architecture, size and selected area electron diffraction (SAED) pattern. A typical TEM image of SNBCs (Figure 2a, 2b) revealed the presence of maximum number of spherical SNBCs. The average diameter of 281 particles measured in TEM was 9.46 ± 6.45 nm. A high resolution TEM (HRTEM) image of SNBCs synthesized at pH 5.0 (Figure 2c) showed the well resolved interference fringe patterns separated by 0.24 nm which corresponded well to the spacing between (111) plane of *fcc* silver crystal (JCPDS. No.01-087-0597). The patterns of SAED (Figure 2d) were indexed according to (111), (200), (220), and (311) reflections of *fcc* silver crystal on the basis of their *d*-spacings of 2.47 A°, 2.13A°, 1.49 A°, and 1.27 A°. The TEM, HRTEM and SAED pattern of SNBCs synthesized at pH 3.0, pH 7.0 and pH 9.0 showed similar pattern as obtained in SNBCs synthesized at pH 5.0 (Additional file 3 Online Resource 3) However, there were distinct differences in their hydrodynamic diameter and zeta potential values.

The zeta potential of SNBCs was measured to know their colloidal stability and the nature of the charge carried in their surface. The average zeta potential of SNBCs, synthesized by extracellular filtrate of pH 5.0, was approximately −21 mV (Additional file 4 Online Resource 4). In spite of this zeta potential value the SNBCs were well stabilized at room temperature as determined by measuring the hydrodynamic diameter by DLS, 30 days after synthesis, with little or no aggregation at all.

**Figure 3:**
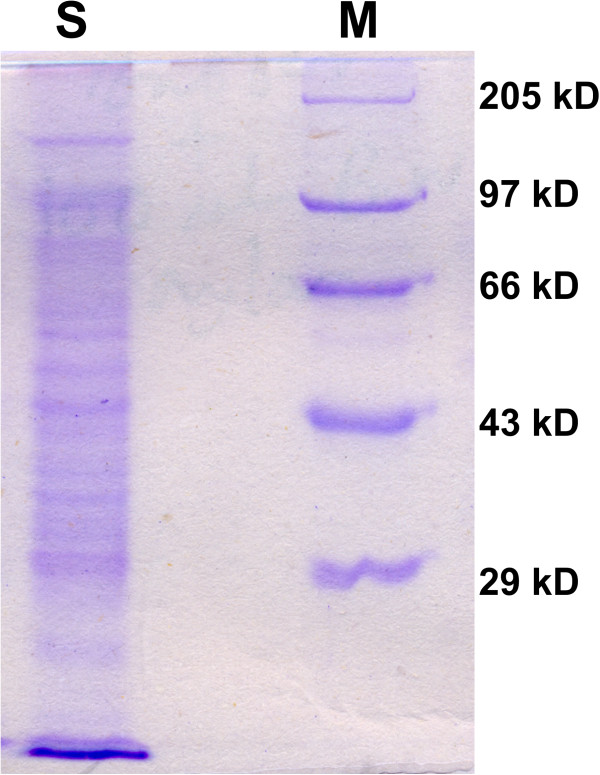
**SDS-PAGE profile of extracellular filtrate.** 20 μg sample protein was loaded in the well and was separated by 10% SDS-PAGE.

**Figure 4:**
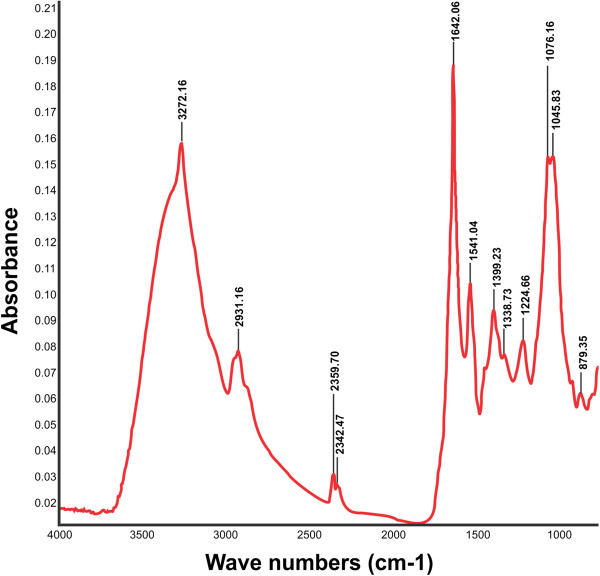
**FT-IR spectra of SNBCs. SNBCs were centrifuged at 10,000 rpm for 10 minutes to remove free proteins or other compounds present in the solution.** The SNBCs then resuspended in double distilled water and again centrifuged. The process was repeated for three times and finally the centrifuged part containg SNBCs were redispersed in double distilled water and subjected to FTIR spectroscopy.

To investigate the reason for stabilization up to 30 days after synthesis of SNBCs, it was further characterized in SDS-PAGE (Figure 3) to find if any proteins were present on the surface of the nanoparticles or not. SDS-PAGE profile of the extracellular filtrate clearly shows the presence of proteins in the filtrate. SNBCs were then analyzed by FT-IR spectroscopy in solid mode to provide further evidence (Figure 4) and it showed both amide I (1642.06 cm^-1^) and amide II (1541.04 cm^-1^) stretching frequencies present in SNBCs ( Caruso et al. 1998).

We have tested the antibacterial activity of SNBCs against *E. coli* and *B. subtilis* and antifungal activity against *S. pombe*. Antimicrobial activity of SNBCs at a concentration of 200 μg/ml was determined by agar diffusion assay (Additional file 5 Online Resource 5). Zone of inhibition determined for *E. coli* by measuring the diameter of bacterial clearance after 24 hour was 3.68 ± 0.1 mm and that for *B. subtilis* was 2.81 ± 0.2 mm. Zone of inhibition determined for *S. pombe* by measuring the diameter of fungal clearance after 24 hour was 1.87 ± 0.15 mm. We have also used 0.5 mM silver nitrate and fungal cell exudates in MilliQ water as control to assess their antimicrobial potency. It was found that silver nitrate showed antimicrobial activity like that of SNBCs. But no zone of inhibition was observed in case of fungal cell exudates in MilliQ water.

The Minimum inhibitory concentrations (MIC) was defined as the lowest silver concentration, which showed no increase in optical density (OD), i.e. no bacterial or fungal growth during 24 hours of inoculation. MIC of SNBCs against *E. coli*, *B. subtilis* and *S. pombe* were represented in Additional file 6 Online Resource 6. In the case of Gram negative bacteria *E. coli* the MIC was 4 μg/ml while for Gram positive bacteria *B. subtilis* the MIC was 8 μg/ml and for the *S. pombe* the value was 8 μg/ml. The minimal concentration of SNBCs which gave rise to plates without bacterial colonies was considered as the minimal bactericidal concentration (MBC). The MBC (or MFC) of SNBCs in growth medium was 8 μg/ml for *E. coli* and 32 μg/ml for *B. subtilis* and 16 μg/ml for *S. pombe* (Additional file 6 Online Resource 6).

**Figure 5:**
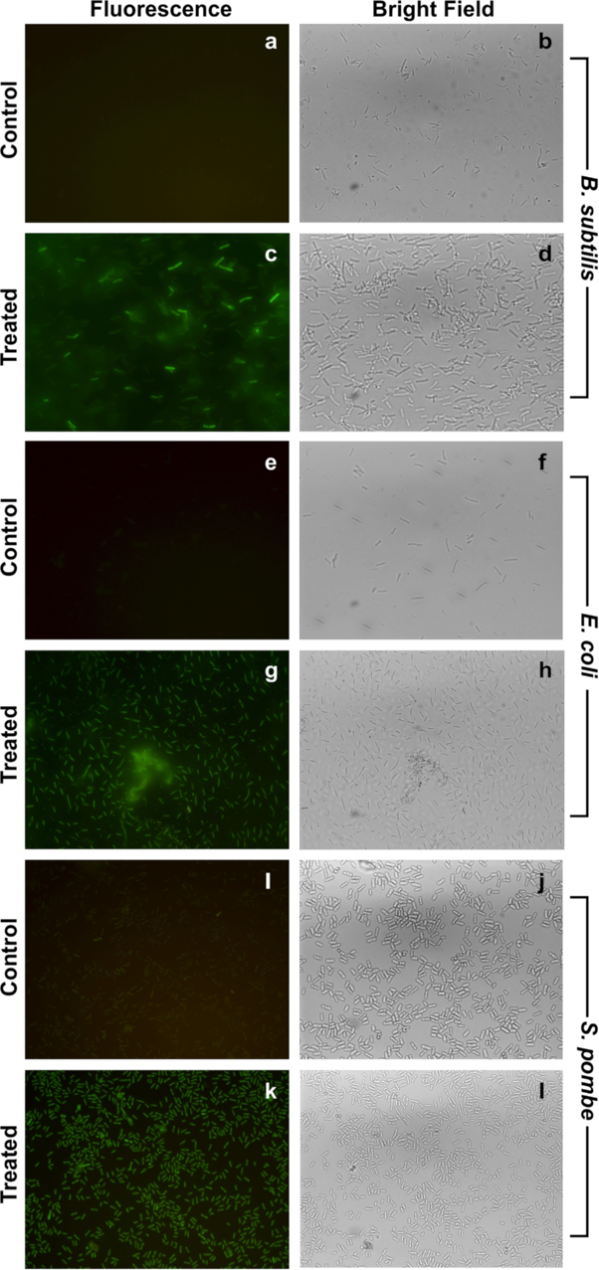
**Generation of reactive oxygen species (ROS) inside of bacteria in response to SNBCs action.** ROS generation was monitored by using 2^′^,7^′^ dichlorodihydrofluorescein diacetate (DCFDA). (**a**) Control *B.s subtilis* fluorescent image. (**b**) Control *B. subtilis* bright field image. (**c**) Treated *B. subtilis* fluorescent image. (**d**) Treated *B. subtilis* bright field image. (**e**) Control *E. coli* fluorescent image. (**f**) Control *E. coli* bright field image (**g**) Treated *E.coli* fluorescent image (**h**) Treated *E.coli* bright field image. (**i**) Control *S. pombe* fluorescent image. (**j**) Control *S. pombe* bright field image. (**k**) Treated *S. pombe* fluorescent image. (**l**) Treated *S. pombe* bright field image.

To investigate the mechanism of action of SNBCs towards its antimicrobial activity, we found reactive oxygen species (ROS) generation inside both bacterial and fungal cells (Figure 5). *E. coli* cells (0.24 O.D. at 590 nm) when treated with SNBCs at a final concentration of 40 μg/ml showed generation of ROS as studied by fluorescence microscopy. The control set showed no ROS generation. Treatment of *B. subtilis* (0.24 O.D. at 590 nm) with SNBCs at a final concentration of 40 μg/ml showed generation of ROS as studied by fluorescence microscopy. The control set showed no ROS generation. *S. pombe* (0.4 OD at 590 nm) when treated with SNBCs at a final concentration of 40 μg/ml also showed generation of ROS as studied by fluorescence microscopy. The control set showed no ROS generation.

## Discussion

In our study SNBCs formed a single population as revealed by DLS analysis in the size range of 20–40 nm. The physical nature of SNBCs as revealed by TEM images that most of the SNBCs were quite spherical in shape with their average diameter of 9.46 ± 6.45 nm. It is true that quantification of Ag (0) is not accurate from the absorbance value of the SPR signal of the SNBCs. Concentration calculation from the molar extinction coefficient value depends on the size of the molecule in a homogeneous population. Although microbial synthesized SNBCs contain a heterogeneous population of different size, still we tried to quantitate the Ag (0) content of SNBCs with an average diameter 9.46 nm and 15.91 nm using the extinction coefficient value of 4.16 × 10^9^ M^-1^ cm^-1^. ( Yguerabide and Yguerabide 1998). It is observed that 15.69 × 10^-2^nM Ag (0) is produced from 0.5 mM AgNO_3_.

Average zeta potential of SNBCs measured to be −21 mV suggesting that some biomolecules capped the surface imparting a net negative charge over it. It could be well assumed that the biomolecules present in extracellular filtrate not only involved in the synthesis but providing the surface coating of silver nanoparticles making them well stable. SDS-PAGE profile of the extracellular filtrate clearly showed the presence of proteins in the filtrate. It is conceivable that the biomolecules present in extracellular filtrate are not only involved in the synthesis but also providing the surface coating of silver nanoparticles making them well stable. So it is possible that some of these proteins and other biomolecules such as chitin, lipid etc. may be present on SNBCs and help to stabilize the SNBCs. It has been shown that silver nanoparticles have affinity for free amine groups or SH-group of cysteine residues of the proteins or through electrostatic attraction of negatively charged carboxylate groups ( Gole et al. 2001). FT-IR analysis of SNBCs again confirmed the presence of biomolecules in the SNBCs.

The ever growing bacterial resistance to antimicrobial agents poses a serious problem in the treatment of infectious diseases as well as in epidemiological practice ( Neu 1992). The surface structures of silver nanoparticles are found to be become important in mediating its antimicrobial activity ( Cho et al. 2005). Thus we wanted to know the antimicrobial property of SNBCs which have outer coating of biomolecules. In this study antibacterial activity of SNBCs was observed against *E. coli* and *B. subtilis* and antifungal activity against *S. pombe*. The zone of inhibition as determined by agar diffusion method for *E. coli* was larger than that for *B. subtilis*. This was most probably due to variation in cell wall composition between gram negative *E. coli* and gram positive *B. subtilis*. It was evident from Additional file 6 Online Resource 6 that both MIC and MBC values were higher for *B. subtilis* than *E. coli*. This may be due to the fact that cell wall structure in *B. subtilis* provided resistance against the diffusion and action of SNBCs.

Generation of reactive oxygen species (ROS) inside both bacterial and fungal cells provides the mechanism of action of SNBCs towards its antimicrobial activity. It is known that majority of nanomaterials such as zinc oxide, carbon nanotubes, and silicon dioxide exert their toxic effects through oxidative stress ( Yang et al. 2008). It is believed that nanoparticle toxicity is multifactorial, where size, shape, surface functionalization and potential to release the corresponding metal ions could play pivotal roles. ROS generation in the presence of SNBCs could be explained by metabolic disturbances as well as other toxicological outcomes. It is also possible that surface oxidation of silver nanoparticle liberates Ag^+^ ions that could amplify the toxicity. Reactions between H_2_O_2_ and silver nanoparticle may be responsible for release of Ag^+^ (Kumar ions *in vivo*^2006^).

A possible chemical reaction involves 

Thus silver ions may be released from SNBCs upon its reaction with H_2_O_2_ which was produced by the action of SNBCs on *E. coli, B. subtilis* and *S. pombe*. Proteomic analysis of the effect of silver ions (Ag^+^) on expression of various proteins in *E. coli* showed a reduction in expression of ribosomal subunit S2, succinyl coenzyme (CoA) synthetase, and maltose transporter ( Yamanaka et al. 2005). It was quite obvious that the reduction in expression of ribosomal subunit S2 impairs the synthesis of proteins, whereas reduction in synthesis of succinyl CoA synthetase and maltose transporter causes suppression of intracellular production of ATP. All these factors were involved in killing mechanism of SNBCs.

In conclusion, the focus of this manuscript was on the antibacterial mechanism of stable silver nanobioconjugate, synthesized by an eco-friendly process. It was found that SNBCs exerted antimicrobial activity towards both Gram positive and Gram negative bacteria and fungus. The MIC and MBC values were higher for Gram positive bacteria (i.e. *B. subtilis*) than Gram negative bacteria (i.e. *E. coli*). We found generation of ROS as mediator of antimicrobial activity of SNBCs.

## Competing interests

The authors declare that they have no competing interests.

## Supplementary Material

Additional file 1 **Online resource 1.** Extracellular synthesis of Silver nano-bioconjugate at pH 5.0. (**a**) Picture of extracellular filtrate. (**b**) Picture of silver nano-bioconjugate. Click here for file

Additional file 2 **Online resource 2.** Measurement of hydrodynamic diameter of SNBCs by Dynamic Light Scattering. Click here for file

Additional file 3 **Online resource 3.** The TEM, HRTEM and SAED pattern of SNBCs synthesized at pH 3.0, pH 7.0 and pH 9.0. TEM image of SNBCs synthesized by extracellular fungal extract of pH 3.0 (**a**); pH 7.0 (**d**) and pH 9.0 (**g**). SAED pattern of SNBCs of pH 3.0 (**b**), pH 7.0 (**e**) and pH 9.0 (**h**) which were indexed according to (111), (200), (220), and (311) reflections of *fcc* silver crystal on the basis of their *d*-spacing. HRTEM image of SNBCs synthesized by extracellular fungal extract of pH 3.0 (**c**); pH 7.0 (**f**) and pH 9.0 (**i**). Click here for file

Additional file 4 **Online resource 4.** Zeta potential of SNBCs. Click here for file

Additional file 5 **Online resource 5.** Antimicrobial activity assay using agar diffusion. Determination of antimicrobial activity of SNBCs, silver nitrate (AgNO3) and fungal cell exudate in agar diffusion assay against *E. coli* and *B. subtilis* and *S. pombe.* The data was represented as mean ± SD. Click here for file

Additional file 6 **Online resource 6.** Minimum inhibitory concentrations, minimal bactericidal concentrations and minimal fungicidal concentrations of SNBCs. Minimum inhibitory concentrations (MIC) of SNBCs were determined against *E.coli*, *B. subtilis* and *S. pombe.* Minimal Bactericidal Concentrations (MBC) of SNBCs were determined against *E.coli* and *B. subtilis* and Minimal Fungicidal Concentrations (MFC) against *S. pombe*. Click here for file

## References

[B1] Bhainsa KC D’Souza SF Extracellular biosynthesis of silver nanoparticles using the fungus *Aspergillus fumigatus* Colloids and Surface B Biointerfaces 2006 47 160 164 10.1016/j.colsurfb.2005.11.026 16420977

[B2] Burda C Chen X Narayanan R EI-Sayed MA Chemistry and properties of nanocrystals of different shapes Chem Rev 2005 105 1025 1102 10.1021/cr030063a 15826010

[B3] Caruso F Furlong DN Ariga K Ichinose I Kunitake T Characterization of Polyelectrolyte-Protein Multilayer Films by Atomic Force Microscopy, Scanning Electron Microscopy, and Fourier Transform Infrared Reflection-Absorption Spectroscopy Langmuir 1998 14 4559 4565 10.1021/la971288h

[B4] Castellano JJ Shafii SM Ko F Donate G Wright TE Mannari RJ Pyne WG Smith DJ Robson MC Comparative evaluation of silver-containing antimicrobial dressings and drugs Int Wound J 2007 4 114 122 10.1111/j.1742-481X.2007.00316.x 17651227 PMC7951235

[B5] Chau CF Wu SH Yen GC The development of regulations for food nanotechnology Trends Food Sci Technol 2007 18 269 280 10.1016/j.tifs.2007.01.007

[B6] Cho KH Park JE Osaka T Park SG The study of antimicrobial activity and preservative effects of nanosilver ingredient Electrochim Acta 2005 51 956 960 10.1016/j.electacta.2005.04.071

[B7] Dror-Ehre A Mamane H Belenkova T Markovich G Adin A Silver nanoparticle- *E. coli* colloidal interaction in water and effect on *E-coli* survival J Colloid Interface Sci 2009 339 521 526 10.1016/j.jcis.2009.07.052 19726047

[B8] Feng QL Wu J Chen GQ Cui FZ Kim TN Kim JO A mechanistic study of the antibacterial effect of silver ions on *Escherichia coli* and *Staphylococcus aureus* J Biomed Mater Res 2000 52 662 668 10.1002/1097-4636(20001215)52:4<662::AID-JBM10>3.0.CO;2-3 11033548

[B9] Gole A Dash C Ramakrishnan V Sainkar SR Mandale AB Rao M Sastry M Pepsin-gold conjugates: preparation, characterization, and enzymatic activity Langmuir 2001 17 1674 1679 10.1021/la001164w

[B10] Gu H Ho PL Tong E Wang L Xu B Presenting vancomycin on nanoparticles to enhance antimicrobial activities Nano Lett 2003 3 1261 1263 10.1021/nl034396z

[B11] Holt KB Bard AJ Interaction of silver (I) ions with the respiratory chain of *Escherichia coli*: an electrochemical and scanning electrochemical microscopy study of the antimicrobial mechanism of micromolar Ag^+^ Biochemistry 2005 44 13214 13223 10.1021/bi0508542 16185089

[B12] Hong B Kai J Ren Y Han J Zou Z Ahn CH Kang KA Highly sensitive rapid, reliable, and automatic cardiovascular disease diagnosis with nanoparticle fluorescence enhancer and MEMS Adv Exp Med Biol 2008 614 265 273 10.1007/978-0-387-74911-2_30 18290337

[B13] Kathiresan K Manivannan S Nabeel MA Dhivya B Studies on silver nanoparticles synthesized by a marine fungus, *Penicillium fellutanum* isolated from coastal mangrove sediment Colloids Surf B Biointerfaces 2009 71 133 137 10.1016/j.colsurfb.2009.01.016 19269142

[B14] Kim KJ Sung WS Moon SK Choi JS Kim JG Lee DG Antifungal effect of silver nanoparticles on dermatophytes J Microbiol Biotechnol 2008 18 1482 1484 18756112

[B15] Klaus T Joerger R Osson E Granqvist CG Silver based crystalline nanoparticles, Microbially fabricated Proc Natl Acad Sci 1999 96 13611 13614 10.1073/pnas.96.24.13611 10570120 PMC24112

[B16] Kumar C Nanomaterials-toxicity, health and environmental issues 2006 Weinheim, Germany: Wiley- VCH Verlag GmbH & Co

[B17] Lee KS EI-Sayed MA Gold and silver nanoparticles in sensing and imaging: sensitivity of plasmon response to size, shape, and metal composition J Phys Chem B 2006 110 19220 19225 10.1021/jp062536y 17004772

[B18] Monali G Jayendra K Avinash I Aniket G Mahendra R Fungus-mediated synthesis of silver nanoparticles and their activity against pathogenic fungi in combination with fluconazole Nanomedicine: Nanotechnology, Biology, and Medicine 2009 5 382 386 10.1016/j.nano.2009.06.005 19616127

[B19] Nair B Pradeep T Coalescence of nanoclusters and formation of submicron crystallites assisted by *Lactobacillus* Strain Cryst Growth des 2002 2 293 298 10.1021/cg0255164

[B20] NCCLS, Approved standards M7–A4 Methods for dilution antimicrobial susceptibility tests for bacteria that grow aerobically 1997 Wayne, PA: National Committee for Clinical Laboratory Standards

[B21] Neu HC The crisis in antibiotic resistance Science 1992 257 1064 1073 10.1126/science.257.5073.1064 1509257

[B22] Nie S Emory SR Probing single molecules and single nanoparticles by surface enhanced Raman scattering Science 1997 275 1102 1106 10.1126/science.275.5303.1102 9027306

[B23] Rai M Yadav A Gade A Silver nanoparticles as a new generation of antimicrobials Biotechnol Adv 2009 27 76 83 10.1016/j.biotechadv.2008.09.002 18854209

[B24] Raveendran P Fu J Wallen SL Completely “green” synthesis and stabilization of metal nanoparticles J Am Chem Soc 2003 125 13940 13941 10.1021/ja029267j 14611213

[B25] Sondi I Salopek-Sondi B Silver nanoparticles as antimicrobial agent: a case study on E. coli as a model for Gram-negative bacteria J Colloid Interface Sci 2004 275 177 182 10.1016/j.jcis.2004.02.012 15158396

[B26] Sukdeb P Yu Kyung T Joon Myong S Does the antibacterial activity of silver nanoparticles depend on the shape of the nanoparticle? a study of the Gram-negative bacterium *Escherichia coli* Appl Environ Microbiol 2007 73 1712 1720 10.1128/AEM.02218-06 17261510 PMC1828795

[B27] Sun RWY Chen R Chung NPY Ho CM Lin CLS Che CM Silver nanoparticles fabricated in HEPES buffer exihibit cytoprotective activities toward HIV-1 infected cells Chem Commun 2005 40 5059 5061 10.1039/b510984a 16220170

[B28] Velev OD Kaler EW In Situ Assembly of Colloidal Particles into Miniaturized Biosensors Langmuir 1999 15 3693 3698 10.1021/la981729c

[B29] Vigneshwaran A Kathe AA Silver-protein (core-shell) nanoparticle production using spent mushroom substrate Langmuir 2007 23 7113 7117 10.1021/la063627p 17518485

[B30] Vigneshwaran N Ashtaputre NM Varadaranjan PV Nachane RP Paralikar KM Balasubramanya RH Biological synthesis of silver nanoparticles using the fungus *Aspergillus flavus* Mater Lett 2007 61 1413 1418 10.1016/j.matlet.2006.07.042

[B31] Vigneshwaran N Kathe AA Varadarajan PV Nachane RP Balasubramanya RH Functional finishing of cotton fabrics using silver nanoparticles J Nanosci Nanotechnol 2007 7 1893 1897 10.1166/jnn.2007.737 17654961

[B32] Vigneshwaran N Nachane RP A novel one-pot green synthesis of stable silver nanoparticle using soluble starch Carbohydrate Res 2006 341 2012 2018 10.1016/j.carres.2006.04.042 16716274

[B33] Wu J Hou SY Ren DC Mather PT Antimicrobial properties of nanostructured hydrogel webs containing silver Biomacromolecules 2009 10 2686 2693 10.1021/bm900620w 19681604

[B34] Xie J Lee JY Wang DIC Ting YP Identification of active biomolecules in the high-yield synthesis of single-crystalline gold nanoplates in algal solutions Small 2007 3 672 682 10.1002/smll.200600612 17299827

[B35] Xu XHN Brownlow WJ Kyriacou SV Wan Q Viola JJ Real-time probing of membrane transport in living microbial cells using single nanoparticle optics and living cell imaging Biochem 2004 43 10400 11043 10.1021/bi036231a 15301539

[B36] Yamanaka M Hara K Kudo J Bactericidal actions of a silver ion solution on *Escherichia coli*, studied by energy-filtering transmission electron microscopy and proteomic analysis Appl Environ Microbiol 2005 71 7589 7593 10.1128/AEM.71.11.7589-7593.2005 16269810 PMC1287701

[B37] Yang H Liu C Yang D Zhang H Xi Z Comparative study of cytotoxicity, oxidative stress and genotoxicity induced by four typical nanomaterials: the role of particle size, shape and composition J Appl Toxicol 2008 29 69 78 18756589 10.1002/jat.1385

[B38] Yguerabide J Yguerabide EE Light-scattering submicroscopic particles as highly fluorescent analogs and their use as tracer labels in clinical and biological applications Ana Biochem 1998 262 137 156 10.1006/abio.1998.2759 9750128

[B39] Zhang L Gu FX Chan JM Wang AZ Langer RS Farokhzad OC Nanoparticles in medicine: therapeutic applications and developments Clin Pharmacol Ther 2008 83 761 769 10.1038/sj.clpt.6100400 17957183

[B40] Zhang Y Peng H Huang W Zhou Y Yan D Facile preparation and characterization of highly antimicrobial colloid Ag or Au nanoparticles J Colloid Interface Sci 2008 325 371 376 10.1016/j.jcis.2008.05.063 18572178

[B41] Zhao G Stevens SE Jr Multiple parameters for the comprehensive evaluation of the susceptibility of *Escherichia coli* to the silver ion BioMetals 1998 11 27 32 10.1023/A:1009253223055 9450315

