# Supplementary material for: An Ecofriendly synthesis of silver nano-bioconjugates by Penicillium citrinum (MTCC9999) and its antimicrobial effect
Source: AMB Express. 2013 Feb 23;3:16. doi: 10.1186/2191-0855-3-16 (PMC3610205; doi:10.1186/2191-0855-3-16)
Supplement: Additional file 1 — Online resource 1. Extracellular synthesis of Silver nano-bioconjugate at pH 5.0. (a) Picture of extracellular filtrate. (b) Picture of silver nano-bioconjugate. [file 2191-0855-3-16-S1.pdf]

Title: An Ecofriendly synthesis of silver nano-bioconjugates by *Penicillium citrinum* (MTCC9999) and its antimicrobial effect

Journal Name: AMB Express

Author Names: Achintya Mohan Goswami, Tuhin Subhra Sarkar and Sanjay Ghosh

Affiliation and Email address of the Corresponding author: Dr. Sanjay Ghosh

Department of Biochemistry, University of Calcutta, 35, Ballygunge Circular Road, Kolkata-700 019, West Bengal, India.

Email: ghoshs71@hotmail.com , sgbioc@caluniv.ac.in

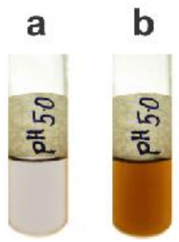

Online Resource 1: Extracellular synthesis of Silver nano-bioconjugate at pH 5.0. (a) Picture of extracellular filtrate. (b) Picture of silver nano-bioconjugate.
