# Supplementary material for: An Ecofriendly synthesis of silver nano-bioconjugates by Penicillium citrinum (MTCC9999) and its antimicrobial effect
Source: AMB Express. 2013 Feb 23;3:16. doi: 10.1186/2191-0855-3-16 (PMC3610205; doi:10.1186/2191-0855-3-16)
Supplement: Additional file 3 — Online resource 3. The TEM, HRTEM and SAED pattern of SNBCs synthesized at pH 3.0, pH 7.0 and pH 9.0. TEM image of SNBCs synthesized by extracellular fungal extract of pH 3.0 (a); pH 7.0 (d) and pH 9.0 (g). SAED pattern of SNBCs of pH 3.0 (b), pH 7.0 (e) and pH 9.0 (h) which were indexed according to (111), (200), (220), and (311) reflections of fcc silver crystal on the basis of their d-spacing. HRTEM image of SNBCs synthesized by extracellular fungal extract of pH 3.0 (c); pH 7.0 (f) and pH 9.0 (i). [file 2191-0855-3-16-S3.pdf]

Title: An Ecofriendly synthesis of silver nano-bioconjugates by *Penicillium citrinum* (MTCC9999) and its antimicrobial effect

Journal Name: AMB Express

Author Names: Achintya Mohan Goswami, Tuhin Subhra Sarkar and Sanjay Ghosh

Affiliation and Email address of the Corresponding author: Dr. Sanjay Ghosh

Department of Biochemistry, University of Calcutta, 35, Ballygunge Circular Road, Kolkata-700 019, West Bengal, India.

Email: ghoshs71@hotmail.com , sgbioc@caluniv.ac.in

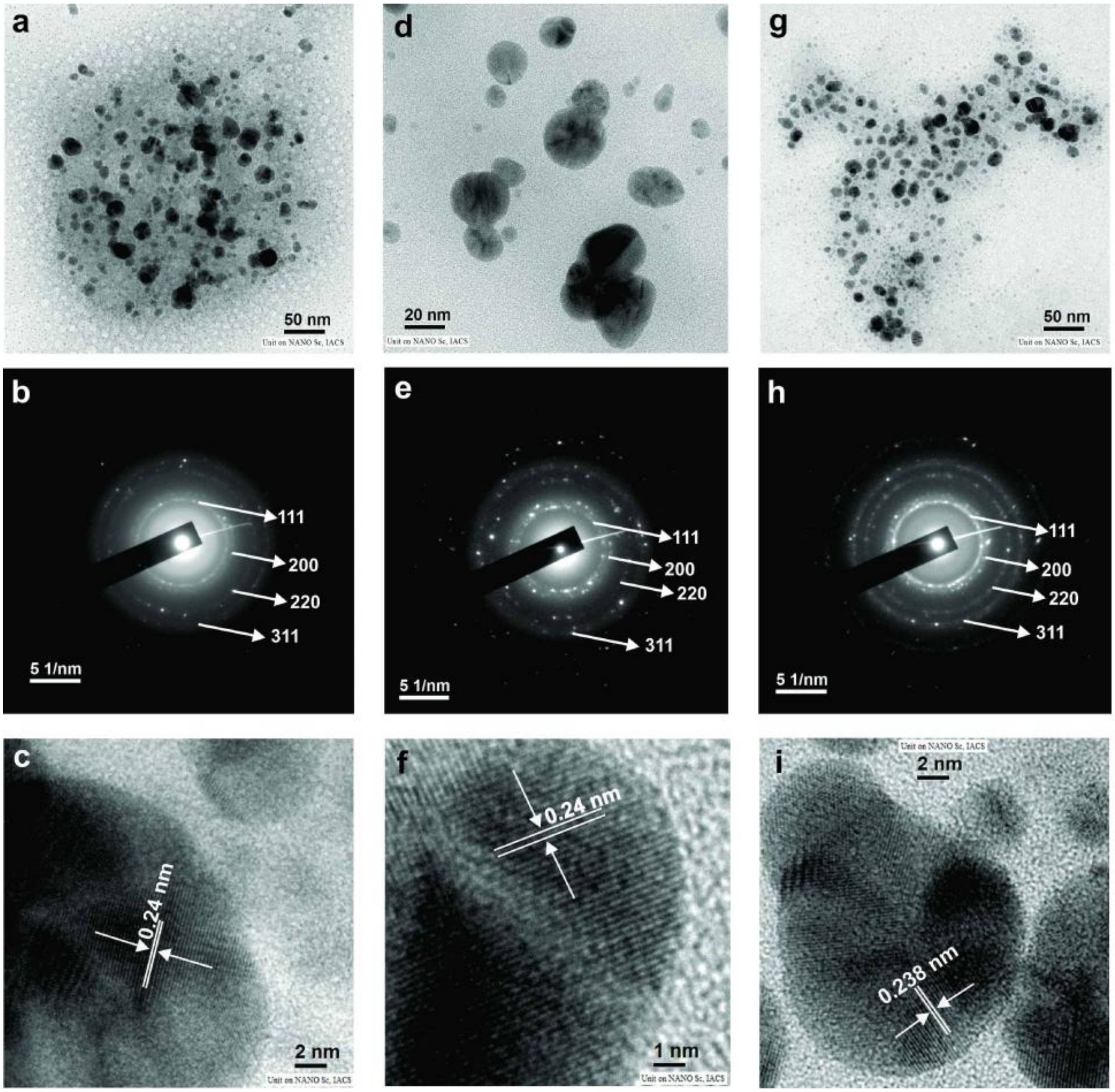

Online Resource 3: The TEM, HRTEM and SAED pattern of SNBCs synthesized at pH 3.0, pH 7.0 and pH 9.0. TEM image of SNBCs synthesized by extracellular fungal extract of pH 3.0 (a); pH 7.0 (d) and pH 9.0 (g). SAED pattern of SNBCs of pH 3.0 (b), pH 7.0 (e) and pH 9.0 (h) which were indexed according to (111), (200), (220), and (311) reflections of *fcc* silver crystal on the basis of their *d*-spacing. HRTEM image of SNBCs synthesized by extracellular fungal extract of pH 3.0 (c); pH 7.0 (f) and pH 9.0 (i).
