# Supplementary material for: An Ecofriendly synthesis of silver nano-bioconjugates by Penicillium citrinum (MTCC9999) and its antimicrobial effect
Source: AMB Express. 2013 Feb 23;3:16. doi: 10.1186/2191-0855-3-16 (PMC3610205; doi:10.1186/2191-0855-3-16)
Supplement: Additional file 5 — Online resource 5. Antimicrobial activity assay using agar diffusion. Determination of antimicrobial activity of SNBCs, silver nitrate (AgNO3) and fungal cell exudate in agar diffusion assay against E. coli and B. subtilis and S. pombe. The data was represented as mean ± SD. [file 2191-0855-3-16-S5.pdf]

Title: An Ecofriendly synthesis of silver nano-bioconjugates by *Penicillium citrinum* (MTCC9999) and its antimicrobial effect

Journal Name: AMB Express

Author Names: Achintya Mohan Goswami, Tuhin Subhra Sarkar and Sanjay Ghosh

Affiliation and Email address of the Corresponding author: Dr. Sanjay Ghosh

Department of Biochemistry, University of Calcutta, 35, Ballygunge Circular Road, Kolkata-700 019, West Bengal, India.

Email: ghoshs71@hotmail.com , sgbioc@caluniv.ac.in

**Strain**	**Zone diameter inhibition in mm**		
	**0.5mM $AgNO_3$**	**200µg/ml SNBCs**	**Fungal cell exudates in MilliQ water**
E. coli	3.58 ± 0.1	3.68 ± 0.1	Not detected
B. subtilis	2.8 ±0.3	2.81± 0.2	Not detected
S. pombe	1.83 ± 0.1	1.87 ± 0.15	Not detected

Online Resource 5: Antimicrobial activity assay using agar diffusion. Determination of antimicrobial activity of SNBCs, silver nitrate (AgNO3) and fungal cell exudate in agar diffusion assay against *E. coli* and *B. subtilis* and *S. pombe.* The data was represented as mean ±SD.
