# Supplementary material for: An Ecofriendly synthesis of silver nano-bioconjugates by Penicillium citrinum (MTCC9999) and its antimicrobial effect
Source: AMB Express. 2013 Feb 23;3:16. doi: 10.1186/2191-0855-3-16 (PMC3610205; doi:10.1186/2191-0855-3-16)
Supplement: Additional file 6 — Online resource 6. Minimum inhibitory concentrations, minimal bactericidal concentrations and minimal fungicidal concentrations of SNBCs. Minimum inhibitory concentrations (MIC) of SNBCs were determined against E.coli, B. subtilis and S. pombe. Minimal Bactericidal Concentrations (MBC) of SNBCs were determined against E.coli and B. subtilis and Minimal Fungicidal Concentrations (MFC) against S. pombe. [file 2191-0855-3-16-S6.pdf]

Title: An Ecofriendly synthesis of silver nano-bioconjugates by *Penicillium citrinum* (MTCC9999) and its antimicrobial effect

Journal Name: AMB Express

Author Names: Achintya Mohan Goswami, Tuhin Subhra Sarkar and Sanjay Ghosh

Affiliation and Email address of the Corresponding author: Dr. Sanjay Ghosh

Department of Biochemistry, University of Calcutta, 35, Ballygunge Circular Road, Kolkata-700 019, West Bengal, India.

Email: ghoshs71@hotmail.com , sgbioc@caluniv.ac.in

Online Resource 6: Minimum inhibitory concentrations, minimal bactericidal concentrations

Strain	MIC of SNBCs	MBC (or MFC) of SNBCs
E. coli	4 µg/ml	8µg/ml
B. subtilis	8/ml	32µg/ml
S. pombe	8/ml	16µg/ml

and minimal fungicidal concentrations of SNBCs. Minimum inhibitory concentrations (MIC) of SNBCs were determined against *E.coli*, *B. subtilis* and *S. pombe.* Minimal Bactericidal Concentrations (MBC) of SNBCs were determined against *E.coli* and *B. subtilis* and Minimal Fungicidal Concentrations (MFC) against *S. pombe*.
